# A randomised controlled trial evaluating two cognitive rehabilitation approaches for cancer survivors with perceived cognitive impairment

**DOI:** 10.1007/s11764-022-01261-5

**Published:** 2022-10-15

**Authors:** Janette L. Vardy, Gregory R. Pond, Melanie L. Bell, Corrinne Renton, Ann Dixon, Haryana M. Dhillon

**Affiliations:** 1 Faculty of Medicine and Health, University of Sydney, Sydney, Australia; 2 Concord Cancer Centre, Concord Repatriation General Hospital, Sydney, Australia; 3 Centre for Medical Psychology & Evidence-Based Decision-Making, University of Sydney, Sydney, Australia; 4 McMaster University, Hamilton, ON Canada; 5 Mel and Enid Zuckerman College of Public Health, University of Arizona, Tucson, AZ USA

**Keywords:** Cancer-related cognitive impairment, Cognitive rehabilitation, Randomised control trial, Cognitive symptoms

## Abstract

**Purpose:**

Up to 70% of survivors report cognitive symptoms after chemotherapy. We compared two cognitive rehabilitation programs to a control group in cancer survivors.

**Methods:**

Study population were adult cancer survivors with cognitive symptoms 6–60 months after adjuvant chemotherapy. Participants randomised to: Attention Process Training (APT), Compensatory Strategy Training (CST), or control group. Active interventions comprised 6–week, 2–h/week small group sessions. Assessments: pre- and post-intervention, 6- and 12-months later. Primary outcome was change in cognitive symptoms (FACT-COG-PCI subscale) between baseline and post-intervention. Secondary endpoints included objective neuropsychological performance, Functional Impact Assessment (FIA), patient-reported outcome measures, and associations. Analyses were on an intention-to-treat basis. Analysis of covariance mixed models were used for continuous outcomes.

**Results:**

Sixty-five participants were randomised (APT *n* = 21; CST *n* = 24; controls *n* = 20): 94% breast cancer, median age 54. Median time since chemotherapy 20.7 months. FACT-COG-PCI, clinical neuropsychological *T*-scores, and FIA improved in all groups over time, but no significant differences between arms. On mean neuropsychological *T*-scores 19/65 (29%) were impaired at baseline; post-intervention impairment controls 31.3%, CST 16.7%, APT 20.0%. On FIA at baseline, nine were impaired; this decreased to three post-intervention (one/group). FACT-COG-PCI was weakly associated with neuropsychological tests (rho = 0.24, *p* = 0.051) at baseline, and had no association with FIA. Neuropsychological total mean *T*-score was moderately positively associated with FIA (rho = 0.37, *p* = 0.003).

**Conclusion:**

There were no significant differences between intervention groups and controls using linear mixed models adjusted for baseline scores.

**Implications for Cancer Survivors:**

Cognitive symptoms and neuropsychological test scores improve over time.

**Supplementary Information:**

The online version contains supplementary material available at 10.1007/s11764-022-01261-5.

## Introduction

Cancer-related cognitive impairment (CRCI) is reported to be the side effect impacting most on survivor’s daily functioning and quality of life (QOL) [1–3]. Up to 70% of cancer survivors who received adjuvant chemotherapy report their memory, attention, and/or concentration are poorer than before diagnosis [1, 4–7].

As assessed by neuropsychological tests, CRCI affects ~ 30% of cancer survivors, with incidence dependent on treatment regimen, time since treatment, method of assessment, and criteria used to define cognitive impairment [1, 8–10]. Cognitive deficits are predominantly in the domains of processing speed, learning and memory, and executive function [11].

Multiple studies have reported poor associations between objective cognitive impairment after chemotherapy and survivors’ self-report of their cognitive function [8, 12]. Self-reported CRCI is moderately strongly associated with fatigue, anxiety, depression, and QOL [6, 13]; with no association generally found between these variables and objective cognitive function [4, 8, 12]. Regardless, both perceived and objective cognitive impairment cause distress to many cancer survivors [1].

Despite how common CRCI is, its impact on everyday functioning is poorly understood. Most cancer survivors reporting cognitive symptoms do not undergo neuropsychological testing, and standard neuropsychological tests assess cognitive function outside everyday contexts; consequently scores may not reflect the practical difficulties cancer survivors describe [14, 15]. The International Cognition and Cancer Task Force (ICCTF) recommend ecologically valid tests be incorporated to evaluate everyday functioning and real-world outcomes [16].

The aetiology of cognitive impairment in cancer patients following chemotherapy is likely multifactorial but remains poorly understood [17]. There is a lack of evidence to guide treatment of CRCI, but there are promising early results for cognitive rehabilitation programs [18].

Cognitive rehabilitation refers to behaviourally oriented interventions designed to improve performance in cognitive and functional domains. The goals are to enhance a person’s capacity to process and interpret information to improve their functional ability [19–21]. Cognitive rehabilitation models generally emphasise one of the following approaches:

- Retraining of a specific cognitive function(s).
- Teaching compensatory techniques to help adaptation to deficits.
- Holistic methods addressing social, emotional, and functional issues related to impairment [22].

The NIH Consensus Development Panel in 1999 found evidence for efficacy of cognitive interventions focusing on attention, memory and executive skills for individuals after traumatic brain injury [19]. Since then cognitive rehabilitation programs have been shown to improve overall cognitive function and goal attainment behaviour [23], as well as memory, problem solving and psychosocial functioning in people with acquired cognitive impairment [24–26]. They work best in people with mild impairment, problems in executive function, and those capable of applying strategic training to real-world demands [27]. There is ongoing debate about the prevalence, extent and nature of cognitive improvements, and whether they are due to improvements in specific skills or reversal of the cognitive deficit.

A systematic review of 19 cognitive rehabilitation studies evaluated 13 intervention programs, either computer-based training or compensatory strategy training in 1124 cancer survivors. All studies reported improvement on at least one self-reported or objective cognitive measure, with 87% of studies (13/15) with control groups, finding improvement in objective measures, particularly memory, executive function, and processing speed [18]. Ten of 16 studies (63%) with longer follow-up found intervention effects persisted at least 2 months later. Effect sizes varied from small to large.

Although cognitive rehabilitation programs are routinely used in other clinical populations with acquired cognitive impairment, they are not routinely offered to cancer survivors with CRCI, and the type of rehabilitation program to provide the most benefit is not known. Our aim was to evaluate two cognitive rehabilitation programs used in non-cancer populations against standard-of-care control in solid tumour survivors self-reporting cognitive impairment after chemotherapy. The cognitive rehabilitation programs were (1) a structured neurocognitive learning program (attention process training [APT]), aimed at improving underlying cognitive deficit; and, (2) psychoeducation and systematic teaching of strategies to compensate for the functional impact of cognitive deficits (compensatory strategy training [CST]). Our a priori hypotheses were that participants randomised to cognitive rehabilitation would: (1) report less impairment; (2) have improved objective neuropsychological performance, better QOL, and less fatigue, anxiety and depression; and (3) have improvement in cognitive symptoms sustained up to 12 months post-intervention.

## Methods

We conducted a longitudinal, multicentre, parallel, unblinded, randomised controlled trial (RCT). All participants gave written, informed consent, and the study had ethical and regulatory approval (approval number: 2019/ETH12998). The trial was prospectively registered with Australian New Zealand Clinical Trials Registry (ACTRN12612001041842).

The study population was initially restricted to breast cancer survivors, who had received at least three cycles of adjuvant chemotherapy, completed any chemotherapy, radiotherapy, and immunotherapy (e.g. trastuzumab) within the last 6–60 months; self-reported cognitive change on the 2-item European Organisation for Research and Treatment of Cancer-Quality of Life Questionnaire-C30 Cognitive Functioning scale (EORTC-CF) [28] as “quite a bit” or greater in one or both domains; and able to read and write English. Hormonal treatment was permitted if commenced > 4 weeks pre-randomisation and unlikely to be ceased within 6 months. One year after the first patient was recruited, the population was expanded to include men or women with any adult onset, non-central nervous system solid tumour to increase recruitment. In addition, the eligibility method of rating cognitive symptoms was amended from the EORTC-CF to the investigator-written Single Item Cognitive Impairment Question “do you think your brain is working as well as it was before your cancer diagnosis?” This change was made as potential participants often self-reported cognitive impairment but not necessarily in the areas covered by the EORTC-CF questions.

Key exclusion criteria included: recurrent or metastatic disease; ECOG performance status ≥ 2; any major pre-existing neurological condition, co-morbidity, psychiatric history, or substance abuse, that could interfere with cognitive performance; prior malignancy within the last 5 years; or previous chemotherapy.

Participants were recruited from nine Sydney hospitals (via referral from clinical staff of survivors reporting cognitive symptoms) or self-referral via the Australian National Breast Cancer Foundation research register (which sent out intermittent emails to registrants informing them of recruiting studies), or our free-call phone number. Participants were randomly allocated (1:1:1) to the APT intervention, CST intervention, or control group. Randomisation was managed centrally using an interactive voice response system and treatment allocation was determined by minimisation, with participants stratified by: institution, primary tumour type (breast cancer vs. other), hormonal treatment (yes/no).

### Intervention groups

Both active interventions comprised a 2-h weekly small group session for 6 weeks, with a behavioural scientist (HD), following detailed manuals. Homework was given weekly to both groups.

Attention process training (APT) was individualised and goal-directed depending on the participant’s deficits and goals, consisting of repetitive computerised exercises designed to practice increasingly difficult attention tasks [15].

Compensatory strategy training (CST) comprised structured sessions with approximately equal time given to compensatory strategies, education and feedback, relaxation and stress management, and psychosocial support.

### Control group

The control group received no active intervention (current standard-of-care); the only contact was for neuropsychological assessments. After the 12-month assessment control participants were able to select participation in the intervention they preferred. They did not receive further follow-up for outcomes.

### Assessments

Subjects were individually evaluated under the guidance of a trained Research Assistant at baseline (before randomisation), after the 6-week intervention, and to assess any sustained improvements 6 and 12 months later. Assessments were initially in person but after April 2020 due to COVID-19 restrictions most were converted to virtual assessments (with functional assessments omitted). Total assessment time was ~4 h with a midway break or could be split into two sessions (Table 1).

**Table 1:** Assessments conducted at baseline, post-intervention, 6 months, and 12 months after intervention

Patient-reported outcome measures	
Variable	Patient-reported outcome measures
Cognitive symptoms	Functional Assessment of Cancer Therapy Cognitive Function version 3 (FACT-COG) questionnaire [35].
	35 items: 4 subdomains: perceived cognitive impairment (PCI) perceived cognitive abilities (PCA) impact of perceived cognitive impairments on quality of life (COG-QOL) comments from others (COG-Other) Higher score equates to fewer symptoms European Organisation for Research and Treatment of Cancer-Quality of Life Questionnaire-C30 Cognitive Functioning scale (EORTC-CF) [28] 2-items Higher score equates to fewer symptoms
Anxiety and depression	Hospital Anxiety Depression Scale (HADS) [38] 7 items: anxiety 7 items: depression Higher score equates to higher symptoms
Quality of life	FACT-General (G) 27 items; 4 Domains: Physical wellbeing Social wellbeing Emotional wellbeing Functional wellbeing higher score equates to fewer symptoms
Fatigue	FACT-Fatigue (F) subscale 13-items higher score equates to fewer symptoms
Physical activity	Active Australia Exercise Questionnaire [41] 9 items
Clinical neuropsychological assessments	
Cognitive domain	**Test**
Premorbid intelligence (baseline only)	Wide Range Achievement Test (WRAT) 3 Reading test
Fluency	Controlled Oral Word Association Test (COWAT)
Executive function	Trail Making Test Part B Wisconsin Card Sorting Test (WCST) (32 item) Stroop Colour and Word
Speed of information processing	Symbol Digit Modalities Test (SDMT) Trail Making Test Part A
Attention working memory	Wechsler Memory Scale (WMS) III Digit Span Wechsler Adult Intelligence Scale (WAIS) III Letter-Number Sequencing WMS-III Spatial Span
Verbal and visual learning	
Verbal:	Hopkins Verbal Learning Test-Revised—(HVLT-R) Brief Visuospatial Memory Test-Revised—(BVMT-R)
Visual	
Motor skills	Grooved pegboard
Functional impact assessment (FIA) [30]	
Domain	Description
Shopping task—from direct assessment of functional status instrument (DAFS) [42]	Participants recall items from a list and select items from a mock grocery store
Basic finances: (DAFS)	Participants recall items from a list and select items from a mock grocery store Participants count money, make change from a $5 note, write a cheque, and balance a cheque account
Advanced finances:	Participants are provided with blank cheques, a bank statement, and calculator; they deposit to a fictional account and pay bills, leaving $100 in the account
Medication Management: a revised version of the Medication management test [43],	Evaluates ability to manage 5 medications
Meal planning:	Participants follow recipes to simulate preparing a simple meal aiming to have three components ready at the same time [30].

### Measures and evaluations

#### Patient-reported outcome measures

Self-reported symptoms of cognition, anxiety/depression, fatigue, QOL and physical activity were evaluated.

#### Neuropsychological assessments

1. Clinical neuropsychological assessments:

Validated neuropsychological tests with normative data available were selected to cover the main domains recommended by ICCTF [29]. Time: 90 min.

(2) Functional impact assessment (FIA):

The FIA was designed to assess performance under ‘real world’ conditions. All tests have been validated in other populations [30], and we piloted them in cancer populations [31–33]. Time: 90 min.

#### Focus group and structured interviews

At the completion of the first APT and CST groups, focus groups were conducted by a staff member not involved in the intervention to obtain participant feedback regarding the intervention and any recommended changes from participants. Individual 10-min structured interviews were conducted by telephone with participants of the second APT group (*n*=4). Focus groups and interviews were recorded, transcribed, and analysed by HD using an interpretive theory approach [34]. Participants from the first two groups were included in the qualitative component to provide an opportunity for refinement of the program if necessary before running additional groups. Feedback suggested both interventions were acceptable and no changes were made to either program.

### Statistical analysis

The primary outcome was change from baseline to post-intervention between each experimental group and the control group in the 20-item perceived cognitive impairments (PCI) subscale of the Functional Assessment of Cancer Therapy Cognitive Function version 3 (FACT-COG) questionnaire [35].

Secondary endpoints were exploratory and hypothesis generating, and included:

Objective neuropsychological performance.

(i) Change from baseline to post-intervention in *T-*scores and a summary score compiled from demographically corrected *T*-scores (age, sex, and education) [36].
(ii) Differences between groups in rates of cognitive impairment

Cognitive impairment was defined using ICCTF recommendations: ≥ 1.5 SD below the normative mean on ≥ 2 tests or ≥ 2 SD on one test. Proportion of participants with cognitive impairment on FIA was based on a functional deficit scale (FDS) ≥ 0.52 [37].

### Patient-reported outcome measures

Subjects were classified as having moderate anxious or depressive symptoms if the Hospital Anxiety Depression Scale (HADS) [38] was > 10/21, and severe for scores > 14/21. Self-reported physical activity results were calculated as time spent in various types and intensity of physical activity per week. Associations between PROMs and neuropsychological test scores were explored using Spearman correlation coefficients.

Analyses were performed on an intention-to-treat basis. Analysis of covariance (ANCOVA) mixed models with baseline mean centering were used to model continuous outcomes while accounting for covariance between repeated measures. Patterns of missing data were considered, but given the small number of missing data, formal testing did not occur, and no patterns of interest were identified. Study data were collected and managed using REDCap electronic data capture tools hosted at the University of Sydney [39, 40]. Analyses were performed in SAS v9.0 (SAS Institute, Cary, NC).

Transcripts from focus groups and interviews were analysed for themes and a coding system developed, and reported in a descriptive, qualitative manner.

#### Sample size

Our initial sample size of 159 participants was calculated to detect a standardised effect size of 0.5 for FACT-COG-PCI for comparing either active treatment groups with the control, with 80% power, assuming 20% dropout, a 2-sided *t*-test, and type I error of 0.05. Our calculation was based upon using baseline measures as covariates (ANCOVA), assuming correlation between baseline and follow-up of 0.6. This gave power to detect a 29% reduction in the proportion with objective neuropsychological impairment between either of the intervention groups and the control group, assuming a control group impairment rate of 40%. Due to slower than expected recruitment, and then recruitment suspension due to COVID-19, the trial was stopped after 65 participants were randomised.

## Results

**Fig. 1:**
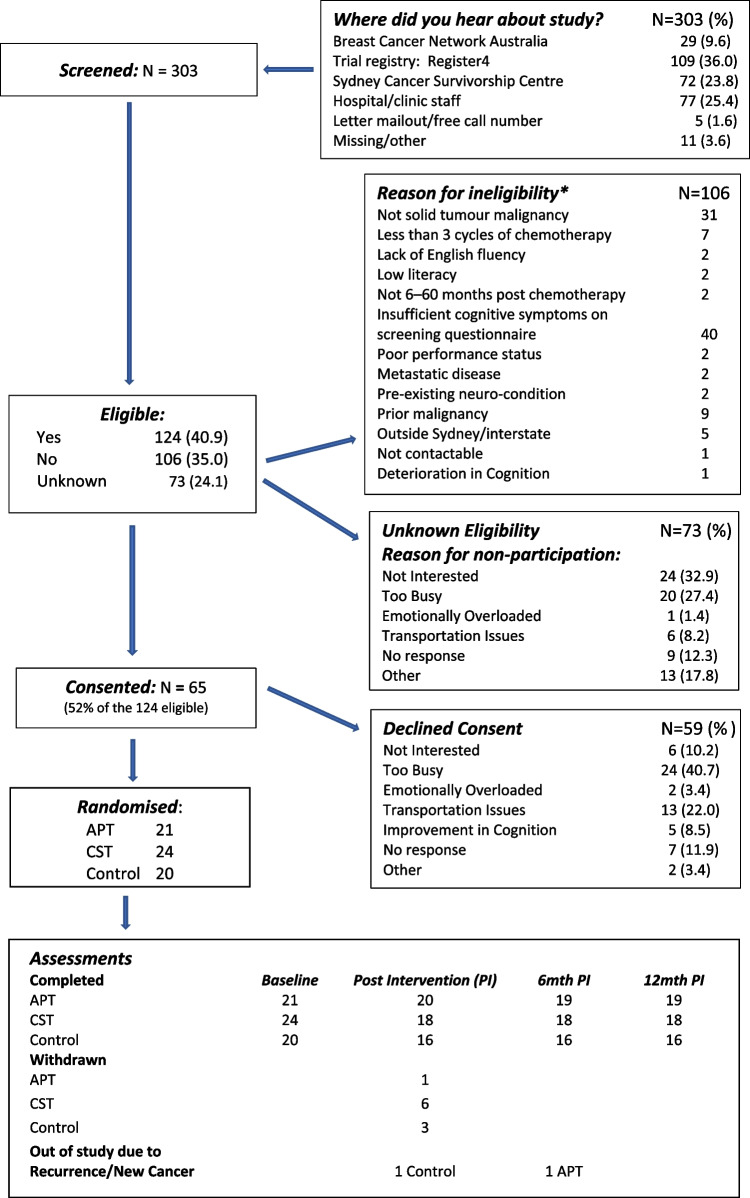
Consort diagram

In total, we screened 303 people; 65 were randomised: APT *n* = 21; CST *n* = 24, and controls *n* = 20. (Fig. 1). Major reasons for eligible people declining to participate were lack of interest and too busy.

For both APT and CST arms, seven groups were held from September 2015 to November 2020.” Group size average was 3/group (range 1–5). This included two people having individual sessions due to COVID restrictions. Five controls, who had completed the study (including 12-month follow-up) attended an active intervention group of their choice.

Ten participants withdrew prior to the post-intervention assessment: APT *n* = 1 (did not complete intervention); CST *n* = 6 (three did not attend any intervention session, one did not complete intervention, two did not attend post-intervention assessment); and three controls did not attend post-intervention assessment. Main reasons for withdrawals were competing commitments, including return to work; not contactable; and, not happy with being in control group. Two participants became ineligible during follow-up due to a recurrence or new cancer: one in APT group before 6-month assessment and one control before the post-intervention assessment.

Participant demographic and clinical characteristics were well-balanced at baseline (Table 2). All but one were female; median age was 54 (range 31–74). Four participants had colorectal cancer and one ovarian cancer. All others had breast cancer, with 78% concurrently on hormonal therapy. The majority were post-menopausal, had English as their primary language, a mean of 14.6 years of education, and excellent performance status. Approximately half had a prior history of depression/anxiety. Median time from completing chemotherapy to baseline assessment was 20.7 months (range 8–60 months), longer in the control arm than the intervention groups. Median time from baseline to intervention was 3.3 months.

**Table 2:** Patient characteristics

		Control	CST	APT
*N*		20	24	21
Age median (range) Mean (SD)		55 (31–72)	52.5 (42–66)	55 (38–74)
		54.8 (11.5)	52.2 (6.1)	56.7 (8.6)
Country of birth *N* (%)	Australia	15 (75.0)	16 (66.7)	14 (66.7)
1^st^ language at home *N* (%)	English	19 (95.0)	21 (87.5)	20 (95.2)
Sex *N* (%)	Female	20 (100)	24 (100)	20 (95.2)
Marital status *N* (%)	Married	16 (80.0)	15 (62.5)	13 (61.9)
	Separated/divorced	3	3	4
	Single	0	5	3
	Widowed	1	1	1
Work status *N* (%)	Full time	7 (35.0)	13 (54.2)	10 (47.6)
	Part time	9 (45.0)	7 (29.2)	7 (33.30
	Retired	2 (10.0)	1 (4.2)	3 (14.3)
	Home care duties	1 (5.0)	2 (8.3)	1 (4.8)
	Other	1 (5.0)	1 (4.2)	0
Years of education mean (SD) (range)		14.7 (2.3)	14.5 (2.7)	14.6 (3.3)
		(10, 18)	(10, 19)	(9, 20)
ECOG performance status N (%)	0	16 (80.0)	21 (87.5)	18 (85.7)
	1	4 (20.0)	3 (12.5)	3 (14.3)
Cancer site *N* (%)	Breast	20 (100)	21 (87.5)	19 (90.5)
	Colorectal	0	2 (8.3)	2 (9.5)
	Gyneacological	0	1 (4.2)	0
Cancer stage *N* (%)	1	1 (5.0)	3 (12.5)	1 (4.8)
	2	8 (40.0)	12 (50.0)	12 (57.1)
	3	8 (40.0)	8 (33.3)	8 (48.1)
	Unknown	3 (15.0)	1 (4.2)	0
Chemotherapy regimen *N* (%)	Anthracycline	1 (5.0)	1 (4.2)	1 (4.8)
	Taxane	4 (20.0)	8 (33.3)	3 (14.3)
	Combination	15 (75.0)	10 (41.7)	14 (66.7)
	5FU + oxaliplatin	0	2 (8.3)	0
	Carboplatin + taxane	0	1 (4.2)	0
	Other	0	2 (8.3)	3 (14.3)
Number of chemotherapy cycles median (range)		9 (4, 16)	6 (4, 16)	8 (3, 16)
Radiotherapy *N* (%)	Yes	14 (70.0)	17 (70.8)	15 (71.4)
Breast cancer patients on hormone therapy *N* (%)	Yes	16/20 (80.0)	15/21 (71.4)	16/19 (84.2)
End chemotherapy to baseline median (range) months		31.0 (8.3, 49.4)	20.7 (8.0, 60.8)	16.1 (8.3, 48.3)
End chemotherapy to intervention median (range) months		-	21.0 (9.5, 53.6)	20.2 (10.0, 53.2)
Completed intervention *N* (%)	Yes	-	20 (83.3)	20 (95)

*SD*, standard deviation; *ECOG*, Eastern Co-operative Oncology Group; *CST*, compensatory strategy training; APT, attention process training

There were no obvious differences between groups in cognitive symptoms, objective neuropsychological scores, FIA scores, QOL, or fatigue at baseline (Supplementary Tables 1, 2). There were higher rates of anxiety and depressive symptoms in both intervention groups.

### Cognitive symptoms

No interaction was observed (*p-*value = 0.45) between intervention group and time period; thus, results were presented for the model without interaction term included (Table 3). No significant difference was observed in the FACT-COG-PCI score between any intervention group (Table 3, Supplementary Table 1). FACT-COG PCI scores were significantly higher at the follow-up time periods compared with baseline.

**Table 3:** Results of ANCOVA mixed model with baseline mean centering for cognitive symptoms (FACT-COG-PCI), *T*-score for neuropsychological test domains, and total functional impact assessment scores

Outcome	Factors	Groups	Estimate (std error)	*p*-value
FACT-COG-PCI	Intercept	Continuous	5.18 (2.54)	0.048
	Baseline FACT-COG-PCI	Continuous	0.87 (0.09)	< 0.001
	Intervention	CST APT Control	− 2.40 (3.33) 3.75 (3.27) Reference	0.15
	Time	Post-intervention 6 months 12 months	Reference 3.98 (1.35) 4.43 (1.35)	0.002
Clinical neuropsychological mean *T*-score	Intercept	Continuous	1.69 (0.86)	0.055
	Baseline mean *T*-score	Continuous	0.98 (0.08)	< 0.001
	Intervention	CST APT Control	− 0.36 (1.15) 0.70 (1.13) Reference	0.83
	Time	Post-intervention 6 months 12 months	Reference 1.44 (0.36) 2.02 (0.36)	< 0.001
Functional impact assessment mean score	Intercept	Continuous	1.95 (0.72)	0.009
	Baseline functional score	Continuous	1.03 (0.07)	< 0.001
	Intervention	CST APT Control	0.14 (0.96) 0.46 (0.94) Reference	0.88
	Time	Post-intervention 6 months 12 months	Reference 1.34 (0.32) 2.05 (0.32)	< 0.001

Interaction effects between intervention and time period were not significant for any model (*p* = 0.45 for FACT-COG-PCI, *p* = 0.61 for clinical cognitive mean *T*-score and *p* = 0.82 for Functional Impact Assessment mean score)

*Std error*, standard error; *Time*, 6 and 12 month refers to assessment at 6- and 12-month post-intervention

*FACT-COG-PCI*, Functional assessment cancer therapy–cognitive function: perceived cognitive impairment; *CST*, compensatory strategy training; *APT*, attention process training

### Neuropsychological results and functional impact assessments

Results for the clinical neuropsychological mean *T*-score and FIA mean score were similar to the FACT-COG PCI (Table 3). No differences between intervention groups were observed, but mean scores increased at post-intervention time points.

Based on our definitions, at baseline 19 participants had objectively assessed cognitive impairment: controls 30%; CST 33.3%, and APT 23.8%. Post-intervention, cognitive impairment rates were controls 31.3%, CST 16.7%, and APT 20% (Table 4). At baseline, nine participants were rated as impaired on the FIA: controls 10%; CST 17.4%, and APT 15.8%. Post-intervention this decreased to three participants: one from each group.

**Table 4:** Number with cognitive deficits on clinical neuropsychological tests by cognitive domain and functional impact assessments (FIA)

		Baseline			Post-intervention			Month 6			Month 12		
		Control	CST	APT	Control	CST	APT	Control	CST	APT	Control	CST	APT
*N*	*N* of deficits (> 1.5 SD)	20	24	21	16	18	20	16	18	19	16	18	19
Fluency	0	19	21	20	15	17	16	15	18	17	14	18	17
	1	0	3	0	0	1	3	0	0	2	1	0	1
	2	1	0	1	1	0	0	1	0	0	1	0	1
	3	0	0	0	0	0	1	0	0	0	0	0	0
Executive function	0	14	18	17	12	16	15	12	13	17	14	14	18
	1	4	4	2	3	1	3	2	5	1	0	4	0
	2	1	2	2	0	0	1	1	0	0	1	4	0
	3	1	0	0	1	0	0	1	0	1	1	0	1
	4	0	0	0	0	1	0	0	0	0	0	0	0
	5	0	0	0	0	0	1	0	0	0	0	0	0
Information processing	0	19	21	20	16	18	18	16	17	19	15	17	18
	1	1	3	1	0	0	1	0	1	0	1	1	0
	2	0	0	0	0	0	1	0	0	0	0	0	1
Attention and working memory	0	20	24	20	16	18	19	16	18	19	16	18	18
	1	0	0	1	0	0	1	0	0	0	0	0	1
	2	0	0	0	0	0	0	0	0	0	0	0	0
Verbal and visual learning	0	12	18	14	13	16	17	16	17	17	15	17	18
	1	7	4	4	3	2	1	0	0	0	0	1	0
	2	0	2	3	0	0	0	0	1	2	1	0	0
	3	0	0	0	0	0	1	0	0	0	0	0	0
	4	1	0	0	0	0	1	0	0	0	0	0	1
Motor skills	0	19	17	15	11	11	18	12	9	14	11	11	12
	1	1	2	4	2	1	0	1	4	3	1	1	1
	2	0	4	1	2	2	0	1	0	0	1	1	0
Total	0	8	12	9	10	13	11	11	11	13	13	12	16
	1	6	4	7	1	2	5	2	4	4	0	3	1
	2	3	4	2	1	1	2	1	1	0	1	3	1
	3	1	1	0	3	1	0	0	2	1	2	0	0
	4	1	0	1	0	0	0	2	0	0	0	0	1
	5	1	1	1	1	0	0	0	0	0	0	0	0
	6	0	2	1	0	1	1	0	0	1	0	0	0
	7	0	0	0	0	0	1	0	0	0	0	0	0
Cognitive deficit (> 1.5 SD on 2 tests or > 2 SD on 1 test)	*N*	6	8	5	5	3	4	3	3	2	3	3	2
	(%) Yes	(30.0)	(33.3)	(23.8)	(31.3)	(16.7)	(20.0)	(18.8)	(16.7)	(10.5)	(18.8)	(16.7)	(10.5)
*N*		20	23	20	15	14	18	14	13	17	13	13	13
FIA shopping deficit	*N* (%) Yes	1 (5.0)	0 (0.0)	1 (5.0)	1 (6.7)	0 (0)	0 (0)	0 (0)	0 (0)	1 (5.9)	0 (0)	0 (0)	1 (7.7)
FIA finances deficit	*N* (%) Yes	5 (25.0)	3 (13.0)	6 (30.0)	6 (40.0)	2 (14.3)	4 (22.2)	3 (21.4)	5 (38.5)	7 (41.2)	5 (38.5)	4 (30.8)	3 (23.1)
FIA medication deficit	*N* (%) Yes	0 (0.0)	4 (17.4)	3 (15.0)	1 (6.7)	2 (14.3)	0 (0)	0 (0)	1 (7.7)	1 (5.9)	1 (7.7)	0 (0)	0 (0)
FIA cooking deficit	*N* (%) Yes	7 (35.0)	7 (30.4)	8 (42.1)	3 (20.0)	3 (21.4)	7 (38.9)	3 (21.4)	4 (30.8)	6 (35.3)	5 (38.5)	3 (23.1)	5 (38.5)
FIA total deficit	*N* (%) Yes	2 (10.0)	4 (17.4)	3 (15.8)	1 (6.7)	1 (7.1)	1 (5.6)	0 (0)	2 (15.4)	3 (17.7)	1 (7.7)	1 (7.7)	0 (0)

*FIA*, functional impact assessment. Total deficit defined as functional deficit score of ≥ 0.52

*SD*, standard deviation

### Associations

FACT-COG-PCI was strongly associated with the other FACT-COG subscores at baseline, particularly perceived cognitive abilities (PCA) (rho = 0.76) and EORTC-CF (rho = 0.73). It was moderately well associated with fatigue, general QOL, anxiety, and depression, with a weak association with total clinical neuropsychological test score (rho = 0.24, *p* = 0.051), and no association with FIA FDS (Table 5). The neuropsychological total mean *T*-score was not associated with anxiety or depression but was moderately positively associated with the FIA FDS (rho = 0.37, *p* = 0.003). The total clinical neuropsychological test scores were moderately strongly associated with the FACT-COG-PCI at the post-intervention and 6- and 12-month assessments (rho 0.38 to 0.42), and with the FACT-COG-PCA, opinion of others sub scores, and EORTC-CF at all time points.

**Table 5:** Spearman correlation coefficients (prob >|*r*| under H0: rho = 0) between patient-reported outcomes and neuropsychological test scores at baseline

	COG-QOL	COG-OTH	COG-PCA	EORTC-CF	FACT-G	Fatigue	Anxiety	Depression	NP-total	FIA FDS
FACT-COG-PCI	0.591	0.525	0.760	0.726	0.258	0.593	− 0.2	− 0.397	0.243	− 0.0
	3	5	0	5	9	4	869	9	0	281
	< .0001	< .0001	< .0001	< .0001	0.0373	< .0001	0.0205	0.0010	0.0511	0.8286
COG-QOL		0.2588	0.3501	0.3768	0.5415	0.6677	− 0.3937	− 0.5301	0.3246	− 0.2214
		0.0374	0.0043	0.0020	< .0001	< .0001	0.0012	< .0001	0.0083	0.0837
COG-OTH			0.390	0.540	0.272	0.258	− 0.2	− 0.286	0.170	− 0.0
			2	0	3	9	239	3	5	146
			0.0013	< .0001	0.0282	0.0373	0.0730	0.0208	0.1744	0.9104
COG-PCA				0.5589	0.4445	0.5641	− 0.2590	− 0.3936	0.1631	0.0026
				< .0001	0.0002	< .0001	0.0373	0.0012	0.1943	0.9841
EORTC-CF					0.3558	0.4077	− 0.2895	− 0.4121	0.2116	− 0.0568
					0.0036	0.0007	0.0194	0.0006	0.0906	0.6611
FACT-G						0.7559	− 0.3748	− 0.6938	0.2180	− 0.2592
						< .0001	0.0021	< .0001	0.0811	0.0419
Fatigue							− 0.3344	− 0.6710	0.2810	− 0.2443
							0.0065	< .0001	0.0234	0.0557
Anxiety								0.5430 < .0001	− 0.0595	0.1503
									0.6380	0.2435
Depression									− 0.1526	0.2261
									0.2250	0.0772
NP-total										− 0.3664 0.0034

Observations *N* = 65 for all tests except functional impact assessment *n* = 62

*FACT-COG*, functional assessment cancer therapy–cognitive function; *PCI*, perceived cognitive impairment (primary endpoint)*PCA*, perceived cognitive abilities; *COG-QOL*, impact of perceived cognitive impairments on quality of life; *COG-other*, comments from others; EORTC-CF, European Organisation for Research and Treatment of Cancer-Quality of Life Questionnaire-C30 Cognitive Functioning; *FACT-G*, FACT-general; *Fatigue*, FACT-Fatigue sub-score; QOL, quality of life

Anxiety and depression symptoms taken from the Hospital Anxiety and Depression Scale

*NP-total*, clinical neuropsychological total mean *T*-score

*FDS-deficit*, functional deficit score derived from functional impact assessment

### Perceptions and impact of the program

Themes identified from two focus groups and four interviews with intervention participants (CTS *n* = 4, APT *n* = 5) included relief cognitive changes were recognised, value of the group, and impact of intervention. Overwhelming, participants discussed how grateful they were their cognitive symptoms were recognised as a side effect of their cancer/treatment, with the CST participants valuing the educational information provided. Participants in both groups emphasised the value of the group setting, noting the importance of the shared experience. Both groups considered the intervention to have had an impact on their daily lives: the APT group described being more aware of their cognition and how they approached activities; CST participants discussed feeling “more at peace” with their cognitive changes.

## Discussion

An improvement was seen in the mean cognitive symptom scores in all groups over time, but no significant differences found between the intervention groups compared with controls for cognitive symptoms, mean total *T*-score on neuropsychological test results, or FIA deficit score, using ANCOVA models adjusted for baseline scores. At baseline the mean FACT-COG-PCI score was 29.6 (range 3–55), well below the score of 60 suggested by van Dyk et a.l as discriminating between CRCI cases and non-cases [44]. At baseline, according to our definition, 29% of participants had cognitive impairment (controls 30%, CST 33.3%, and APT 23.8%), with rates decreasing after the intervention in the two intervention groups (controls 31.3%, CST 16.7%, and APT 20%). Consistent with the literature the main cognitive domains affected were verbal and visual learning, and executive function. Motor skills were also impacted but likely due to chemotherapy-induced peripheral neuropathy affecting fine motor tasks as assessed by the grooved pegboard. At baseline, nine participants were rated impaired on the FIA, which decreased to three participants (one in each group) after the intervention. Some of the improvements in the neuropsychological tests, particularly the FIA which does not have alternative versions, are likely due to practice effects.

Our findings are consistent with other studies employing similar interventions. Dos Santos [45] completed a three-arm RCT (*n* = 167 survivors) comparing a computerised training program led by a neuropsychologist, to two active control groups (home-based cognitive exercises or phone follow-up). They found no significant difference between the groups for the primary endpoint of improvement on the FACT-COG-PCI, but a trend for greater improvement in the active group (75% vs. 55–57%). A significant difference was seen in improvement in working memory in the active group.

Similarly, our previous RCT comparing a computer training program to standard-of-care in 242 survivors found improvement in cognitive symptoms, anxiety/depression, stress, and fatigue, and improved QOL sustained 6 months later, but no significant difference in neuropsychological test results [46].

Determining whether cognitive rehabilitation interventions are generalisable to everyday activities could provide important information but few studies have included functional tests performed under ‘real world’ conditions [47]. Our pilot work in breast cancer survivors [31] found global functional deficits in 13%, with at least moderate deficits on cooking tasks in 34% and financial tasks 13%. However, functional assessments in colorectal cancer survivors 6–12 years after diagnosis detected no significant difference in global functional deficit scores compared to a non-cancer control group [33]. In the current study, deficits were most common in the cooking, financial, and medication tasks. Interestingly, we found a moderate association between the neuropsychological total score and the FIA FDS (rho = 0.37, *p* = 0.003), but not between the FACT-COG-PCI and FIA.

Two other small pilot studies that included functional tests found no difference for intervention or time effects but both were underpowered [48, 49]. Limitations of available functional assessments include ceiling effects, and for repeated measures equivalent alternative versions to reduce practice effect.

The qualitative findings from our study suggest differential impacts from the treatment approaches, and tailoring interventions to the specific priorities of individuals should be considered in future research. Our experience with two RCT for CRCI suggests online home-based programs with timing flexible to suit participant’s lifestyles, and may be more feasible for cancer survivors. There is a clear need to understand the components of interventions most impactful for participants, to enable shorter and/or greater tailoring to support stepped care in CRCI. Other suggestions for future research include a comparison between online computer-based training and face-to-face group sessions such as used here to compare efficacy, and development of functional tests more suitable for repeat testing to better determine impact of CRCI on everyday activities and any improvement after cognitive rehabilitation programs.

Strengths of our study include the use of two active interventions and a control group, to determine whether improvement was due to the intervention, practice effect, and/or improvement over time. There were limitations to our study, particularly the smaller than planned sample size due to recruitment difficulties compounded by the COVID-19 pandemic, which meant we were only able to detect larger than planned differences for comparing either active treatment group with the control group. COVID-19 restrictions also necessitated a change in follow-up assessments to online, omission of the FIA, and two participants having their sessions run as individual rather than group sessions. Time between baseline assessment and the intervention was longer than planned waiting for sufficient numbers to run the groups. Staff and participants were not blinded to allocation. Focus groups and interviews were limited to the first two groups and may not reflect the experience of later groups. Most participants were breast cancer survivors so results may not be generalisable to other tumour types or to men.

## Conclusions

Although the literature suggests a benefit to cognitive rehabilitation programs for cancer survivors experiencing cognitive difficulties, we found no significant differences between intervention groups and controls using ANCOVA models adjusted for baseline scores. There was improvement in cognitive symptoms and neuropsychological test scores over time in all groups, with a non-significant trend for greater reduction in proportion of participants with impairment in the intervention groups post-treatment.

## Supplementary Information

Below is the link to the electronic supplementary material.

Supplementary file1 (DOCX 42 KB)

## Data Availability

The datasets generated during and/or analysed during the current study are available from the corresponding author on reasonable request.
