# Supplementary material for: A randomised controlled trial evaluating two cognitive rehabilitation approaches for cancer survivors with perceived cognitive impairment
Source: J Cancer Surviv. 2022 Oct 15;17(6):1583–95. doi: 10.1007/s11764-022-01261-5 (PMC9568997; doi:10.1007/s11764-022-01261-5)
Supplement: Supplementary file 1 — Supplementary file1 (DOCX 42 KB) [file 11764_2022_1261_MOESM1_ESM.docx]

**Supplementary Table 1. Mean (SD) scores for patient reported outcome measures for each group at each timepoint**

|  |  | **Control** | **CST** | **APT** |
| --- | --- | --- | --- | --- |
| **Baseline** | | | | |
| **N** |  | 20 | 24 | 21 |
| **FACT-COG** | PCI  PCA  QOL  Other | 32.7 (12.4)  12.6 (4.6)  10.2 (3.2)  13.6 (2.2) | 27.7 (15.4)  11.0 (4.8)  8.5 (5.1)  12.5 (3.7) | 29.0 (15.3)  13.1 (4.5)  7.7 (4.5)  12.1 (4.6) |
| **EORTC-CF** | Cognitive Functioning | 50.8 (21.9) | 40.3 (26.4) | 52.4 (26.5) |
| **FACT-G**  **QOL** | FACT-G-total score  Domains:  Physical wellbeing  Social wellbeing  Emotional wellbeing  Functional wellbeing | 79.3 (16.9)  20.9 (5.1)  21.5 (5.8)  17.5 (4.5)  19.4 (5.0) | 75.1 (16.3)  20.7 (5.2)  19.9 (6.0)  17.0 (3.2)  17.5 (6.1) | 75.1 (16.2)  18.6 (6.4)  20.8 (6.0)  16.7 (4.9)  19.0 (5.3) |
| **Fatigue** | FACT-Fatigue subscale | 38.9 (9.3) | 36.0 (12.8) | 34.1 (12.6) |
| **HADS*** | Anxiety  Depression | 5.3 (2.4)  5.1 (2.4) | 9.0 (3.4)  7.8 (4.8) | 7.9 (4.2)  6.4 (3.9) |
|  | N (%) Moderate Anxiety  Severe Anxiety  Moderate Depression  Severe Depression | 0 (0)  0 (0)  0 (0)  0 (0) | 10 (41.7)  1 (4.2)  6 (25.0)  3 (12.5) | 6 (28.6)  2 (9.5)  4 (19.1)  0 (0) |
| **Exercise** | Time (minutes)/week  Walking time/week  Vigorous intensity/week  Moderate intensity/week | 508.8 (436.0)  172.0 (117.9)  137.3 (18.4.4)  62.3 (116.9) | 365.8 (320.6)  214.3 (182.0)  56.5 (11.3)  38.5 (80.3) | 470.0 (449.6)  248.8 (17.4)  101.2 (156.5)  18.8 (39.6) |
| **Post-Intervention** | | | | |
| **N** |  | 16 | 18 | 20 |
| **FACT-COG** | PCI  QOL  Other  PCA | 39.3 (16.4)  10.4 (3.5)  13.5 (3.0)  15.2 (5.6) | 31.8 (18.0)  9.3 (5.3)  12.9 (3.3)  11.9 (5.4) | 36.4 (17.4)  10.2 (5.4)  13.5 (3.5)  14.7 (6.6) |
| **EORTC-CF** | Cognitive Functioning | 58.3 (20.2) | 49.1 (28.9) | 62.5 (27.0) |
| **FACT QOL** | FACT-G-total score  Domains:  Physical wellbeing  Social wellbeing  Emotional wellbeing  Functional wellbeing | 81.8 (15.2)  21.6 (4.5)  23.1 (4.4)  18.1 (3.9)  19.9 (4.9) | 74.2 (18.0)  20.9 (5.0)  18.3 (6.1)  17.8 (3.2)  17.2 (6.8) | 80.1 (15.6)  20.4 (6.1)  22.1 (5.7)  17.2 (4.5)  20.5 (5.2) |
| **Fatigue** | FACT-Fatigue subscale | 39.4 (10.0) | 36.4 (11.4) | 38.9 (11.6) |
| **HADS** | Anxiety  Depression | 5.8 (3.1)  5.5 (3.2) | 8.6 (3.4)  8.6 (4.9) | 7.6 (4.0)  5.6 (4.3) |
|  | N (%) Moderate Anxiety  Severe Anxiety  Moderate Depression  Severe Depression | 0 (0)  0 (0)  1 (6.3)  0 (0) | 5 (27.8)  1 (5.6)  7 (38.9)  2 (11.1) | 7 (35.0)  0 (0)  3 (15.0)  0 (0) |
| **Exercise** | Time (minutes)  Walking time/week  Vigorous intensity/week  Moderate intensity/week | 410.3 (296.0)  196.3 (159.8)  55.9 (61.4)  93.7 (39.0) | 432.8 (264.8)  191.7 (206.3)  82.5 (89.4)  76.1 (110.7) | 536.3 (729.2)  261.5 (256.0) 112.6 (223.0)  36.6 (56.5) |
| **Month 6** | | | | |
| **N** |  | 16 | 18 | 19 |
| **FACT-COG** | PCI  COG-QOL  COG-Other  PCA | 39.7 (18.0)  11.1 (4.4)  13.3 (2.8)  14.2 (5.5) | 37.2 (17.1)  9.4 (4.5)  13.6 (3.2)  12.1 (6.5) | 42.4 (15.8)  10.9 (4.2)  13.9 (2.2)  15.6 (5.8) |
| **EORTC-CF** | Cognitive Functioning | 58.3 (25.8) | 56.5 (31.4) | 65.8 (24.5) |
| **FACT-G**  **QOL** | FACT-G-total score  Domains:  Physical wellbeing  Social wellbeing  Emotional wellbeing  Functional wellbeing | 82.0 (17.6)  21.6 (5.3)  22.1 (5.3)  18.6 (3.1)  19.6 (.6) | 74.1 (17.8)  21.6 (5.7)  17.5 (7.3)  17.9 (2.9)  17.1 (7.0) | 78.9 (14.2)  19.8 (5.9)  20.5 (6.6)  18.2 (2.9)  20.4 (5.2) |
| **Fatigue** | FACT-Fatigue subscale | 40.9 (11.0) | 39.0 (10.6) | 37.2 (11.4) |
| **HADS** | Anxiety  Depression | 5.9 (4.5)  5.1 (3.3) | 8.1 (2.9)  8.7 (5.1) | 6.5 (3.9)  5.7 (4.2) |
|  | N (%) Moderate Anxiety  Severe Anxiety  Modererate Depression  Severe Depression | 2 (12.5)  1 (6.3)  1 (6.3)  0 (0) | 4 (22.2)  0 (0)  8 (44.4)  3 (16.7) | 3 (15.8)  1 (5.3)  4 (21.1)  0 (0) |
| **Exercise** | Time (minutes)  Walking time/week  Vigorous intensity/week  Moderate intensity/week | 392.5 (303.7)  188.8 (172.7)  70.6 (77.8)  62.5 (89.8) | 467.6 (351.0)  213.9 (178.9)  106.4 (130.7)  65.3 (73.7) | 422.1 (425.1)  226.1 (218.8)  79.2 (105.5)  37.6 (72.3) |
| **Month 12** | | | | |
| **N** |  | 16 | 18 | 19 |
| **FACT-COG** | PCI  QOL  Other  PCA | 40.5 (22.5)  11.9 (3.3)  14.3 (2.7)  15.2 (5.3) | 37.3 (17.2)  9.7 (5.3)  13.7 (2.8)  13.3 (6.2) | 42.8 (15.0)  11.6 (3.7)  14.1 (2.2)  16.9 (6.2) |
| **EORTC-CF** | Cognitive Functioning | 62.5 (26.2) | 54.6 (32.2) | 71.1 (24.7) |
| **FACT-G**  **QOL** | FACT-G-total score  Domains:  Physical wellbeing  Social wellbeing  Emotional wellbeing  Functional wellbeing | 83.0 (15.0)  21.6 (4.0)  23.0 (4.6)  18.5 (4.6)  19.9 (4.1) | 76.4 (17.2)  22.0 (5.1)  18.7 (6.4)  18.2 (2.7)  17.5 (6.5) | 80.3 (11.5)  21.0 (4.5)  21.7 (5.3)  18.9 (2.6)  21.4 (4.5) |
| **Fatigue** | FACT-Fatigue subscale | 40.1 (11.1) | 39.7 (11.4) | 40.4 (10.2 ) |
| **HADS** | Anxiety  Depression | 5.1 (3.8)  4.8 (3.6) | 7.7 (2.6)  8.1 (4.9) | 5.9 (3.0)  4.2 (3.5) |
|  | N (%) Moder. Anxiety  Severe Anxiety  Modererate Depression  Severe Depression | 1 (6.3)  0 (0)  1 (6.3)  0 (0) | 3 (16.7)  0 (0)  6 (33.3)  3 (16.7) | 1 (5.3)  0 (0)  1 (5.3)  0 (0) |
| **Exercise** | Time (minutes)  Walking time/week  Vigorous intensity/week  Moderate intensity/week | 394.6 (314.5)  180.3 (145.0)  84.4 (93.1)  45.5 (86.7) | 395.0 (258.4)  178.9 (118.1)  68.1 (101.2)  80.0 (116.1) | 479.7 (536.5)  228.0 (255.7)  103.5 (168.7)  44.7 (68.3) |

CST = Compensatory Strategy Training

APT = Attention Process Training

FACT-COG = Functional Assessment Cancer Therapy- Cognitive function:

PCI = perceived cognitive impairment (primary endpoint)

PCA = perceived cognitive abilities

COG-QOL = impact of perceived cognitive impairments on quality of life

COG-Other = comments from others

EORTC-CF = European Organisation for Research and Treatment of Cancer-Quality of Life Questionnaire-C30 Cognitive Functioning scale

Note: FACT and EORTC-CF = higher score equates to less symptoms.

FACT-G= FACT General

QOL = quality of life

HADS = Hospital Anxiety and Depression Scale

*Score of >10 = moderate anxiety or depression; score of >14 = severe

Exercise- measured using the Active Australia questionnaire.

**Supplementary Table 2. Mean (SD) T-scores for clinical neuropsychological tests and mean raw scores and deficit scores for Functional Impact Assessments**

|  |  | **Control** | **CST** | **APT** |
| --- | --- | --- | --- | --- |
| **Baseline** | | | | |
| **N** |  | 20 | 24 | 21 |
| **Cognitive Test** | WRAT3  COWAT Total Letter  COWAT Category animal  COWAT Written Fluency Total  Trail Making B  WCST Perseverative Errors  Stroop Word Score  Stroop Color Score  Stroop Color-Word Score  Symbol Digit  Trail Making Test A  Digit Span  Letter Number Sequence  Spatial Span Total  HVLT Total Recall  HVLT Delayed Recall  BVMT Total Recall  BVMT Delayed Recall  Grooved Pegboard DH  Grooved Pegboard NDH | 56.0 (4.8)  49.7 (9.9)  49.7 (7.6)  51.0 (12.6)  53.0 (8.6)  49.6 (10.1)  44.6 (14.5)  44.8 (9.8)  50.5 (8.1)  54.9 (11.5)  54.2 (7.7)  54.6 (8.7)  55.5 (9.6)  56.8 (8.5)  47.6 (10.2)  47.4 (11.7)  46.2 (13.3)  53.5 (11.1)  47.2 (8.5)  44.3 (7.5) | 54.3 (5.4)  52.7 (10.5)  53.4 (11.0)  50.6 (10.1)  54.6 (10.0)  46.8 (9.8)  49.0 (10.2)  45.8 (9.7)  51.9 (8.1)  54.6 (13.2)  58.3 (11.1) 54.2 (8.2)  59.5 (10.4)  55.0 (9.8)  50.0 (10.0  49.5 (11.6)  55.0 (13.0)  57.8 (10.5)  46.6 (12.3)  42.0 (10.1) | 56.2 (4.3)  48.7 (9.1)  49.5 (7.7)  50.3 (9.2)  53.1 (11.3)  48.0 (8.3)  46.9 (11.7)  45.7 (9.9)  48.1 (7.5)  55.5 (9.6)  54.4 (10.0)  53.6 (7.8)  58.5 (9.8)  54.6 (9.5)  43.1 (8.5)  44.3 (9.9)  50.3 (8.7)  56.0 (8.9)  42.1 (6.1)  42.7 (7.1) |
| **Domain** | Premorbid Intelligence  Fluency  Executive Function  Information Processing  Attention Working Memory  Verbal & Visual Learning  Motor Skills | 56.0 (4.8)  50.1 (8.6)  48.5 (7.3)  54.5 (8.1)  55.6 (7.2)  48.7 (8.6)  45.8 (7.6) | 54.3 (5.4)  52.2 (8.6)  49.6 (6.2)  56.5 (10.7)  56.2 (7.3)  53.0 (8.7)  43.3 (11.0) | 56.2 (4.3)  49.5 (7.4)  48.4 (6.2)  53.7 (10.8)  55.6 (5.6)  48.4 (5.8)  42.4 (5.8) |
| **Total** | Mean T-Score^ | 51.5 (5.7) | 53.5 (6.6) | 51.1 (4.8) |
|  |  |  |  |  |
| **Functional Impact Assessment** | Shopping Total  Shopping Deficit  Finances Total  Finances Deficit  Medication Total  Medication Deficit  Cooking Total  Cooking Deficit | 17.0 (1.9)  0.1 (0.4)  33.7 (1.7)  0.4 (0.7)  15.0 (1.0)  0.0 (0.0)  26.2 (3.0)  0.4 (0.5) | 17.4 (1.3)  0.0 (0.0)  33.4 (2.4)  0.4 (1.2)  14.4 (2.6)  0.3 (0.8)  26.8 (2.6)  0.3 (0.5) | 16.7 (1.8)  0.1 (0.2)  33.2 (1.6)  0.4 (0.6)  14.5 (1.9)  0.2 (0.5)  25.8 (3.8)  0.6 (1.0) |
|  | Total Raw Score (/101)  Total Deficit Score^#^ | 91.8 (5.5)  0.2 (0.3) | 92.1 (5.7)  0.3 (0.5) | 88.9 (7.7)  0.3 (0.4) |
| **Post-Intervention** | | | | |
| **N** |  | 16 | 18 | 20 |
| **Test** | COWAT Total Letter  COWAT Category animal  COWAT Written Fluency Total  Trail Making B  WCST Perseverative Errors  Stroop Word Score  Stroop Color Score  Stroop Color-Word Score  Symbol Digit  Trail Making Test A  Digit Span  Letter Number Sequence  Spatial Span Total  HVLT Total Recall  HVLT Delayed Recall  BVMT Total Recall  BVMT Delayed Recall  Grooved Pegboard DH  Grooved Pegboard NDH | 53.3 (10.4)  53.1 (9.6)  54.1 (12.6)  55.0 (9.3)  53.4 (16.0)  46.3 (12.2)  46.1 (11.0) 52.6 (9.7)  57.9 (11.8)  57.2 (8.6)  51.8 (7.0)  58.3 (10.2)  56.5 (8.6)  55.3 (8.7)  49.3 (9.9)  57.3 (13.6)  56.8 (11.7)  44.4 (10.8)  44.8 (11.0) | 54.6 (9.3)  53.9 (9.3)  57.2 (12.6)  56.4 (11.2)  48.6 (8.0)  51.1 (9.6)  47.7 (10.6)  55.9 (9.2)  59.6 (11.3)  59.4 (11.1)  54.2 (9.7)  56.9 (9.0)  57.9 (8.2)  55.4 (7.1)  51.9 (5.7)  57.7 (11.3)  57.6 (8.4)  46.8 (10.4)  44.7 (13.6) | 51.9 (10.6)  49.8 (12.3)  55.2 (13.1)  53.6 (9.8)  53.0 (15.4)  45.0 (14.4)  45.5 (12.4)  49.1 (10.9)  54.1 (16.7)  55.0 (11.6)  55.0 (10.1)  60.1 (9.6)  56.4 (8.9)  52.7 (11.2)  50.3 (10.9)  55.2 (10.6)  56.1 (11.6)  46.0 (8.7)  45.7 (7.6) |
| **Domain** | Fluency  Executive Function  Information Processing  Attention Working Mem  Verbal & Visual Learning  Motor Skills | 53.5 (9.5)  50.7 (8.1)  57.6 (8.8)  54.3 (6.9)  54.6 (7.9)  44.6 (10.2) | 55.2 (9.0)  51.9 (7.2)  59.5 (10.1)  56.4 (6.9)  55.7 (6.1)  45.8 (11.1) | 52.3 (10.2)  49.1 (9.5)  54.5 (11.5)  57.1 (6.9)  53.6 (9.8)  45.8 (6.7) |
| **Total** | Mean T-Score | 54.2 (6.1) | 55.7 (5.9) | 53.3 (8.1) |
|  |  |  |  |  |
| **Functional Impact Assessment** | Shopping Total  Shopping Deficit  Finances Total  Finances Deficit  Medication Total  Medication Deficit  Cooking Total  Cooking Deficit | 17.1 (2.4)  0.1 (0.5)  32.7 (2.4)  0.7 (1.3)  15.4 (1.3)  0.1 (0.3)  27.1 (2.4)  0.2 (0.4) | 17.9 (1.5)  0.0 (0.0)  33.5 (1.2)  0.2 (0.6)  15.0 (1.5)  0.1 (0.4)  27.4 (2.3)  0.2 (0.4) | 17.9 (1.7)  0.0 (0.0)  33.4 (1.8)  0.3 (0.7)  15.6 (0.6)  0.0 (0.0)  26.7 (3.1)  0.4 (0.5) |
|  | Total Raw Score (/101)  Total Deficit Score | 92.2 (5.1)  0.3 (0.4) | 93.9 (4.6)  0.1 (0.2) | 93.6 (4.3)  0.2 (0.2) |
| **Month 6** | | | | |
| **N** |  | 16 | 18 | 19 |
| **Test** | COWAT Total Letter  COWAT Category animal  COWAT Written Fluency Total  Trail Making B  WCST Perseverative Errors  Stroop Word Score  Stroop Color Score  Stroop Color-Word Score  Symbol Digit  Trail Making Test A  Digit Span  Letter Number Sequence  Spatial Span Total  HVLT Total Recall  HVLT Delayed Recall  BVMT Total Recall  BVMT Delayed Recall  Grooved Pegboard DH  Grooved Pegboard NDH | 55.8 (10.6)  53.4 (12.0)  58.3 (14.2)  56.4 (9.9)  57.2 (16.0)  45.5 (13.7)  45.7 (12.9)  51.3 (11.6)  55.7 (10.9)  58.8 (8.3)  52.4 (8.3)  55.9 (9.2)  58.3 (9.7)  57.0 (9.5)  55.6 (8.4)  54.5 (9.9)  56.9 (9.3)  49.7 (10.5)  48.6 (9.4) | 57.8 (10.6)  56.9 (8.9)  57.0 (10.2)  59.2 (9.7)  48.9 (14.0)  52.4 (9.4)  47.8 (10.6)  57.2 (11.0)  61.7 (11.3)  66.6 (11.4)  55.8 (9.2)  59.4 (8.2)  59.0 (10.5)  57.5 (6.1)  54.7 (5.9)  56.3 (12.0) 56.7 (9.5)  48.4 (11.1)  44.5 (13.5) | 54.9 (10.1)  53.6 (11.3)  54.7 (10.6)  54.1 (10.7)  53.5 (11.9)  47.2 (11.6)  48.3 (11.8)  50.6 (9.6)  58.1 (11.7)  58.4 (9.9)  56.7 (11.0)  59.2 (8.7)  58.1 (10.5)  50.8 (10.8)  51.4 (11.6)  55.6 (12.6)  57.2 (7.6)  45.5 (8.3)  45.1 (9.9) |
| **Domain** | Fluency  Executive Function  Information Processing  Attention Working Mem  Verbal & Visual Learning  Motor Skills | 55.8 (11.2)  51.2 (9.3)  57.3 (8.5)  54.3 (8.0)  56.0 (6.7)  49.2 (8.8) | 57.2 (8.0)  53.1 (7.5)  64.1 (10.2)  58.1 (6.5)  56.3 (7.1)  46.5 (11.5) | 54.4 (8.6)  50.7 (8.1)  58.3 (9.8)  58.0 (7.3)  53.7 (9.2)  45.3 (8.2) |
| **Total** | Mean T-Score | 54.9 (6.6) | 57.8 (5.1) | 55.0 (7.0) |
|  |  |  |  |  |
| **Functional Impact Assessment** | Shopping Total  Shopping Deficit  Finances Total  Finances Deficit  Medication Total  Medication Deficit  Cooking Total  Cooking Deficit | 18.4 (1.6)  0.0 (0.0)  33.8 (1.7)  0.3 (0.6)  15.5 (0.8)  0.0 (0.0)  26.9 (1.9)  0.2 (0.4) | 18.2 (1.2)  0.0 (0.0)  32.5 (2.6)  0.7 (1.4)  14.8 (1.6)  0.1 (0.3)  26.0 (4.0)  0.5 (1.0) | 17.1 (2.0)  0.1 (0.5)  33.1 (1.9)  0.5 (0.6)  15.3 (1.1)  0.1 (0.2)  25.8 (2.0)  0.4 (0.5) |
|  | Total Raw Score (/101)  Total Deficit Score | 94.5 (3.3)  0.1 (0.2) | 91.5 (6.9)  0.3 (0.5) | 91.2 (4.8)  0.3 (0.3) |
| **Month 12** | | | | |
| **N** |  | 16 | 18 | 19 |
| **Test** | COWAT Total Letter  COWAT Category animal  COWAT Written Fluency Total  Trail Making B  WCST Perseverative Errors  Stroop Word Score  Stroop Color Score  Stroop Color-Word Score  Symbol Digit  Trail Making Test A  Digit Span  Letter Number Sequence  Spatial Span Total  HVLT Total Recall  HVLT Delayed Recall  BVMT Total Recall  BVMT Delayed Recall  Grooved Pegboard DH  Grooved Pegboard NDH | 55.2 (12.1)  54.8 (9.2)  59.1 (14.9)  60.6 (8.5)  55.4 (12.1)  46.8 (13.5)  47.0 (12.2)  53.8 (11.7)  59.3 (14.6)  60.9 (8.6)  56.1 (10.3)  61.2 (10.4)  56.1 (10.6)  57.9 (11.0)  54.9 (9.7)  53.1 (9.2)  55.8 (7.9)  50.5 (12.3)  46.2 (9.9) | 57.3 (10.8)  58.0 (9.8)  59.9 (11.0)  62.8 (11.3)  50.2 (10.6)  51.8 (8.3)  49.2 (10.7)  56.2 (9.8)  62.0 (11.6)  63.9 (13.0) 54.0 (8.2)  59.5 (9.0)  58.7 (8.9)  57.2 (8.0) 54.7 (7.0) 56.3 (9.5)  55.8 (7.7)  48.5 (10.4)  47.3 (12.4) | 56.4 (11.4)  53.3 (10.7)  55.2 (13.3)  56.0 (8.4)  54.9 (12.6)  47.5 (12.1)  45.9 (12.8)  49.6 (10.5)  56.2 (12.9)  57.5 (12.6)  60.3 (11.3)  61.2 (10.0) 56.7 (10.3)  55.2 (10.5)  53.4 (9.8)  56.6 (9.7)  57.6 (8.8)  45.7 (9.3)  44.5 (6.8) |
| **Domain** | Fluency  Executive Function  Information Processing  Attention Working Mem  Verbal & Visual Learning  Motor Skills | 56.3 (11.0)  52.7 (8.4)  60.1 (11.2)  56.6 (9.0)  55.4 (6.9)  48.3 (10.4) | 58.4 (8.6)  54.0 (6.8)  62.9 (11.0) 57.4 (7.1)  56.0 (6.4)  47.9 (10.6) | 54.9 (10.4)  50.8 (7.9)  56.8 (11.8)  59.4 (8.5)  55.7 (9.0)  45.1 (7.3) |
| **Total** | Mean T-Score | 56.2 (7.1) | 57.7 (5.9) | 55.5 (8.1) |
|  |  |  |  |  |
| **Functional Impact Assessment** | Shopping Total  Shopping Deficit  Finances Total  Finances Deficit  Medication Total  Medication Deficit  Cooking Total  Cooking Deficit | 18.2 (1.6)  0.0 (0.0)  32.8 (2.4)  0.7 (1.2)  15.1 (1.5)  0.1 (0.3)  26.2 (3.3)  0.4 (0.5) | 18.2 (1.4)  0.0 (0.0)  32.8 (2.4)  0.6 (1.4)  15.2 (1.2)  0.0 (0.0)  26.4 (2.7)  0.2 (0.4) | 16.9 (1.9)  0.1 (0.3)  33.2 (1.9)  0.3 (0.6)  15.2 (0.9)  0.0 (0.0)  25.9 (1.9)  0.4 (0.5) |
|  | Total Raw Score (/101)  Total Deficit Score | 92.2 (5.3)  0.3 (0.3) | 92.6 (5.8)  0.2 (0.4) | 91.2 (4.5)  0.2 (0.1) |

WRAT-3= Wide Range Achievement Test 3 Reading test

Controlled Oral Word Association (COWAT) = Controlled Oral Word Association Test

Wisconsin Card sorting test =WCST

Symbol Digit Modalities Test

Stroop= Stroop Colour and Word

Digit span =Wechsler Memory Scale (WMS) III Digit Span

Letter number sequence = Wechsler Adult Intelligence Scale (WAIS) III Letter-Number Sequencing

Spatial span = WMS-III SpatialSpan

HVLT-R = Hopkins Verbal Learning Test-Revised –(HVLT-R)

BVMT-R = Brief Visuospatial Memory Test-Revised – (BVMT-R)

Grooved Pegboard DH = dominant hand, NDH = non-dominant hand

Mean T-score = mean score of cognitive domains of fluency, executive function, information processing, attention working memory, and verbal/visual learning.
